# Evaluating Treatment Strategies in Advanced Endometrial Cancer: Primary Cytoreductive Surgery Versus Neoadjuvant Chemotherapy Followed by Interval Debulking Surgery—A Ten-Year Single-Centre Experience

**DOI:** 10.1155/ogi/7202848

**Published:** 2025-09-02

**Authors:** Mohamed Abdelwanis Mohamed Abdelaziz, Ahmed Mohamed, Siddesh Prabhulingam, Ambreen Yaseen, Khaled Sabrah, Fatini Hussin, Riyam Aldulaimi, Hazem Elsheikh, Ashu Loona, Irshad Soomro, Ketankumar Gajjar, Benjamin Wormald

**Affiliations:** ^1^Department of Gynaecological Oncology, Nottingham University Hospitals NHS Trust, City Campus, Nottingham NG5 1PB, UK; ^2^Department of Obstetrics and Gynaecology, United Lincolnshire Hospitals, Lincoln County Hospital, Lincoln LN2 5QY, UK; ^3^Pathology Department, Nottingham University Hospitals NHS Trust, City Campus, Nottingham NG5 1PB, UK

**Keywords:** advanced endometrial cancer, cytoreductive surgery, NACT-IDS, neoadjuvant chemotherapy, treatment outcomes

## Abstract

**Introduction/Background:** Management of advanced endometrial cancer (EC) presents a significant therapeutic challenge, with ongoing debate regarding optimal treatment sequencing. Primary cytoreductive surgeries (PCSs) with adjuvant therapy and neoadjuvant chemotherapy followed by interval Debulking surgery (NACT-IDS) are both employed as treatment strategies. This study analyses outcomes of both treatment strategies in Nottingham University Hospitals Cancer Centre.

**Methodology:** We conducted a retrospective cohort analysis of patients with advanced EC (FIGO Stages III-IV) treated at our centre between 2013 and 2023. Patients who received either PCS with adjuvant therapy or neoadjuvant chemotherapy followed by interval Debulking surgery (NACT-IDS) are included in the study. Data collection included demographic characteristics, treatment approaches, surgical parameters, and outcome measures. Primary outcomes were progression-free survival (PFS) and overall survival (OS). Secondary outcomes included perioperative outcomes and recurrence patterns.

**Results:** Treatment pathways included NACT-IDS (*n* = 8) and PCS with adjuvant therapy (*n* = 57). Stage IV disease was notably more prevalent in patients who received NACT-IDS therapy compared to the PCS group (75.0% versus 5.3%, *p* < 0.001). Analysis revealed a PFS duration of 18.5 months for NACT-IDS patients, whilst PCS patients demonstrated a longer duration of 35.5 months (HR 1.18, 95% CI: 0.56–2.48, *p*=0.328). Median OS was 22.0 months in the NACT-IDS group versus 41.0 months in the PCS group (HR 1.35, 95% CI: 0.64–2.83, *p*=0.145). Mean operative time was longer in the NACT-IDS group (239.7 vs 165.5 min, *p*=0.209). All NACT-IDS procedures were performed via open laparotomy compared to 49.1% in the PCS group (*p* < 0.001). Hospital stay was significantly longer in the NACT-IDS group (median 8 vs 3 days, *p*=0.036). Radiotherapy was administered to 25.0% (*n* = 2) of NACT-IDS patients and 59.6% (*n* = 34) of PCS patients. Recurrence rates were higher in the NACT-IDS group, 37.5%, compared to 33.3% in the PCS patients (*p*=0.823).

**Conclusion:** This comprehensive analysis provides valuable insights into treatment outcomes and surgical parameters for advanced EC. Whilst the small sample size of the NACT-IDS cohort limits the ability to draw definitive conclusions, the study provides meaningful evidence that can inform clinical decision-making. The findings lay important groundwork for future prospective, multicentre studies aimed at optimising patient selection and treatment sequencing in this challenging disease.

## 1. Introduction

Endometrial cancer (EC) is a prevalent gynaecological malignancy in developed nations, with increasing incidence amongst postmenopausal women [1]. Whilst early-stage EC typically has a favourable prognosis, advanced-stage disease (FIGO Stages III-IV) presents significant therapeutic challenges and poor outcomes [2]. Traditionally, advanced EC management involved primary cytoreductive surgery (PCS) followed by adjuvant therapy [3]. However, for patients with extensive disease or compromised performance status, neoadjuvant chemotherapy followed by interval Debulking surgery (NACT-IDS) has emerged as a potential alternative approach [4].

Emerging research indicates that NACT-IDS may provide benefits for carefully selected patient populations, particularly those with substantial disease burden or complex medical conditions [5]. The potential benefits include tumour downstaging, improved resectability, and reduced surgical morbidity [6]. However, there remains limited evidence directly comparing outcomes between PCS and NACT-IDS approaches in advanced EC, with most existing data extrapolated from ovarian cancer studies [7]. The role of various prognostic factors, including residual disease status, FIGO stage, and histological subtype, also requires further investigation in the context of different treatment strategies [8].

The varying practices across institutions and the lack of standardised selection criteria for NACT-IDS versus PCS highlight the need for more comprehensive analyses of treatment outcomes [9]. Additionally, the impact of residual disease and histological subtypes on survival outcomes needs better understanding to optimise patient selection and treatment sequencing [10].

### 1.1. Aim of Work

The primary objective of this study was to analyse and compare the clinical outcomes, survival rates, and perioperative characteristics between patients treated with NACT-IDS versus PCS with adjuvant therapy in advanced EC at Nottingham University Hospitals (NHS)Cancer Centre. Secondary objectives included evaluation of surgical outcomes and perioperative morbidity, assessment of chemotherapy response patterns, analysis of prognostic factors affecting survival outcomes and comparison of recurrence patterns between treatment approaches.

## 2. Methodology

### 2.1. Study Design and Population

This single-centre retrospective cohort study was conducted at NHS Cancer Centre, United Kingdom, analysing patients treated between January 2013 and December 2023. The study included 65 patients with advanced EC, divided into two groups: The NACT-IDS group (*n* = 8) and the PCS with adjuvant therapy group (*n* = 57). Eligible patients included women aged 18 years or older with histologically confirmed advanced EC (FIGO Stages III-IV) who received complete treatment at our institution. Patients with early-stage disease (FIGO Stages I-II), nonendometrioid histology without endometrial component, treatment initiated at other institutions, or incomplete clinical or follow-up data were excluded from the study.

### 2.2. Ethical Considerations

This study was conducted as a service evaluation project and was formally registered as an audit with NHS Trust (Project number 24-319C). In accordance with UK Health Research Authority guidelines for service evaluation projects, specific patient consent and ethical approval were not required. All data were collected and analysed in a fully anonymised format with no patient identifiers to ensure confidentiality. The study was conducted in compliance with institutional protocols for clinical audit and service evaluation, adhering to principles of data protection and patient privacy. Data handling and storage followed NHS information governance protocols.

### 2.3. Data Collection and Variables

Data collection covered comprehensive demographic and clinical information, including age at diagnosis, menopausal status, performance status, and medical comorbidities. Tumour characteristics were documented, including FIGO stage, histologic subtype and grade, location of metastatic disease and molecular subtype where available. Treatment details were recorded for both groups: for the NACT-IDS group, this included chemotherapy regimen, number of cycles, response to NACT, and interval Debulking surgical procedures; for the PCS group, primary surgical procedures, adjuvant chemotherapy details and response to treatment were documented. Surgical parameters were collected for all patients, including operative time, estimated blood loss, transfusion requirements, extent of cytoreduction, residual disease status, intraoperative complications and length of hospital stay.

### 2.4. Surgical Procedures and Staging

All patients were staged according to the FIGO 2009 staging classification system. Surgical procedures were performed by experienced gynaecological oncologists following standardised institutional protocols.

Staging Procedures: All patients underwent systematic surgical staging including total hysterectomy and bilateral salpingo-oophorectomy. Lymph node assessment varied by disease extent and patient factors. Sentinel lymph node mapping was not routinely utilised during our study period. Extended cytoreductive procedures were performed as clinically indicated. A modified Aletti surgical complexity score was retrospectively applied to all cases:

• Score 1 (low complexity): Hysterectomy, bilateral salpingo-oophorectomy, ±pelvic/para-aortic lymphadenectomy
• Score 2 (intermediate complexity): Score 1 procedures + omentectomy, appendectomy or limited peritonectomy
• Score 3 (high complexity): Score 2 procedures + bowel resection, diaphragmatic resection or extensive peritonectomy [11].

Histopathological Confirmation: All cases had histological confirmation by experienced gynaecological pathologists. Immunohistochemistry was performed selectively based on morphological features, including oestrogen receptor, progesterone receptor, p53, and mismatch repair proteins (MLH1, MSH2, MSH6, and PMS2) where clinically indicated.

### 2.5. Outcome Measures

The study assessed two primary outcome measures: progression-free survival (PFS), measuring the time from treatment initiation to disease progression or death, and overall survival (OS), tracking the duration from treatment initiation to death from any cause. Secondary outcomes included perioperative outcomes (surgical complications, hospital stay duration and recovery time), treatment response rates and patterns of recurrence (local recurrence and distant metastasis). The quality of residual disease was categorised as either optimal (< 1 cm) or suboptimal (≥ 1 cm).

### 2.6. Statistical Analysis

Statistical analysis was performed using IBM SPSS Statistics Version 26.0 and R software Version 4.1.0. Descriptive statistics presented continuous variables as mean/median with standard deviation (SD)/range and categorical variables as frequencies with percentages. Survival analyses utilised Kaplan–Meier curves, with group differences assessed by log-rank test. Cox proportional hazards models conducted multivariate analysis, providing hazard ratios with 95% confidence intervals. Statistical comparisons were performed based on variables type: for categorical data, either the chi-square or Fisher's exact test was used; for continuous data, Student's *t*-test was employed when the data was normally distributed, whilst the Mann–Whitney *U* test was used for non-normally distributed data. Multivariate analysis included Cox regression for survival outcomes and logistic regression for binary outcomes, adjusting for age, stage, histology, and residual disease. GraphPad Prism version 9.0 generated graphs and figures. Two-sided *p* values less than 0.05 were considered statistically significant.

## 3. Results

### 3.1. Patient Demographics and Cancer Characteristics

In the demographic analysis, the median age was similar between groups (68.5 vs 65.2 years), with both groups predominantly composed of postmenopausal patients. The NACT-IDS group exhibited a higher proportion of patients with poorer performance status (37.5% vs 15.8% PS 2-3) and higher comorbidity rates (75.0% vs 63.2%). Regarding cancer characteristics, there was a significant difference in stage distribution (*p* < 0.001), with the NACT-IDS group having a substantially higher proportion of Stage IV disease (75.0% vs 5.3%). Histological differences were also notable, with endometrioid histology being more common in the PCS group (73.7% vs 50.0%), whilst serous and clear cell histologies were more frequent in the NACT-IDS group (Tables 1 and 2).

### 3.2. Survival Outcomes Analysis

**Figure 1:**
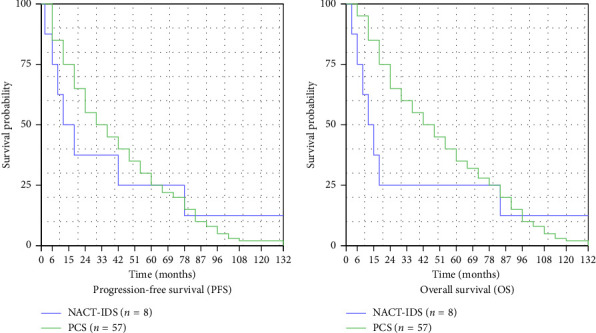
Survival analysis in NACT-IDS and PCS groups (Kaplan–Meier curves).

Survival outcome analysis between the NACT-IDS and PCS groups revealed distinct patterns. The NACT-IDS group (*n* = 8) showed shorter median survival times compared to the PCS group (*n* = 57), a finding contextualised by the significantly higher proportion of Stage IV disease in the NACT-IDS cohort (75.0% vs 5.3%, *p* < 0.001). Median PFS was 18.5 months in the NACT-IDS group versus 35.5 months in the PCS group (HR 1.18, 95% CI: 0.56–2.48, *p*=0.328). Median OS was 22.0 months in the NACT-IDS group compared to 41.0 months in the PCS group (HR 1.35, 95% CI: 0.64–2.83, *p*=0.145). Despite not reaching statistical significance, likely due to the small NACT-IDS sample size, the results suggest a trend toward better survival outcomes in the PCS group (Table 3, Figure 1).

### 3.3. Key Findings

1. PFS Analysis:
  The PFS evaluation revealed a 17-month shorter median in the NACT-IDS group. Despite this difference, statistical significance was not achieved (*p*=0.328), with the hazard ratio indicating only a modest increase in progression risk.
2. OS Findings:
  OS analysis showed a 19-month shorter median in the NACT-IDS group. The difference did not reach statistical significance (*p*=0.145), but the hazard ratio suggested a trend toward increased mortality risk.
3. Recurrence Patterns:
  Recurrence rates were comparable between groups (37.5% vs 33.3%), with similar local and distant recurrence distribution. No statistically significant differences in recurrence patterns were detected.

### 3.4. Surgical Outcomes Analysis

All patients were staged according to the FIGO 2009 staging classification system. Comprehensive analysis of surgical outcomes revealed significant differences between the NACT-IDS and PCS groups in surgical approach, complexity and procedural requirements (Tables 4, 5 and 6).

All NACT-IDS procedures were performed via open laparotomy (100% vs 49.1% in the PCS group, *p* < 0.001), reflecting the complexity of advanced disease in this cohort. Simple total hysterectomy was performed in 98.5% of cases overall, with radical hysterectomy reserved for patients with clinical or radiological evidence of cervical involvement (1 case in the PCS group). The NACT-IDS group demonstrated more extensive lymph node assessment, with 75.0% undergoing both pelvic and para-aortic lymphadenectomy compared to only 10.5% in the PCS group (*p* < 0.001). Among patients who underwent lymphadenectomy, systematic dissection was more commonly performed in the NACT-IDS group (75.0% vs 38.6%, *p*=0.046). Sentinel lymph node mapping was utilized exclusively in the PCS group (7.0% vs 0%). Total omentectomy was significantly more frequent in the NACT-IDS group (62.5% vs 3.5%, *p* < 0.001), reflecting the more extensive cytoreductive approach required for advanced disease.

The NACT-IDS group demonstrated significantly higher surgical complexity scores (mean 2.1 ± 0.8 vs 1.6 ± 0.7, *p*=0.045). Additional surgical procedures were more frequently required in the NACT-IDS group, including appendicectomy (25.0% vs 1.8%, *p*=0.028) and peritonectomy (25.0% vs 3.5%, *p*=0.048).

**Figure 2:**
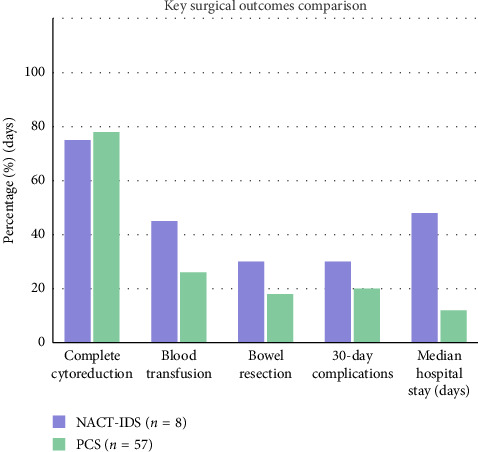
Surgical outcomes of NACT-IDS and PCS groups.

The NACT-IDS group demonstrated longer operative times (mean 239.7 vs 165.5 min, *p*=0.209) and extended hospital stays (median 8 vs 3 days, *p*=0.036). Intraoperative blood loss was comparable between groups, though transfusion requirements were higher in the NACT-IDS cohort (37.5% vs 22.8%, *p*=0.324). Complete cytoreduction (no gross residual disease) was achieved in 62.5% of NACT-IDS patients compared to 70.2% of PCS patients (*p*=0.648). The NACT-IDS group experienced a higher rate of postoperative complications (25.0% vs 15.8%, *p*=0.497), though this difference did not reach statistical significance. These findings suggest that whilst NACT-IDS procedures were generally more complex and time-consuming, they achieved comparable surgical outcomes in terms of residual disease (Figure 2).

### 3.5. Chemotherapy and Treatment Response Analysis

**Figure 3:**
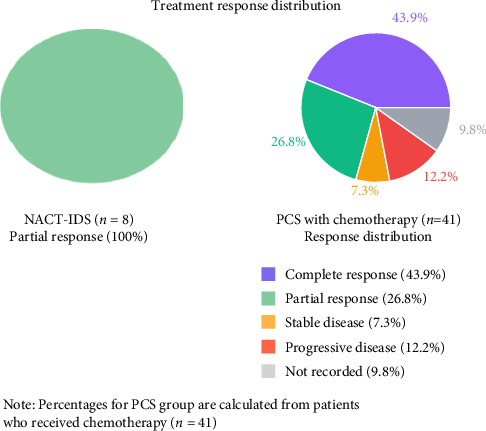
Treatment response distribution in both groups.

In the NACT-IDS group (*n* = 8), all patients received chemotherapy, with the carboplatin and paclitaxel combination being the predominant regimen (87.5%, *n* = 7), whilst one patient received combined chemoradiotherapy (12.5%). The median number of neoadjuvant chemotherapy cycles was 4 (range 3–6). Treatment response assessment showed partial response in all patients (100%, *n* = 8). In the PCS group (*n* = 57), 41 patients (71.9%) received adjuvant chemotherapy, whilst 13 patients (22.8%) did not receive chemotherapy due to patient choice, poor performance status, or death, and 3 patients (5.3%) had no recorded data. Among those who received chemotherapy in the PCS group (*n* = 41), carboplatin-paclitaxel was the most common regimen (61.0%, *n* = 25), with a median of 4 cycles (range 3–8). Treatment response in this group showed complete response in 43.9% (*n* = 18), partial response in 26.8% (*n* = 11), stable disease in 7.3% (*n* = 3), and progressive disease in 12.2% (*n* = 5), whilst 9.8% (*n* = 4) had no recorded response data (Table 7, Figure 3).

### 3.6. Radiotherapy Utilization Analysis

Regarding additional treatment, radiotherapy utilization was higher in the PCS group (59.6%, *n* = 34) compared to the NACT-IDS group (25.0%, *n* = 2), though this difference was not statistically significant (*p*=0.1252). In the PCS group, 6 patients (10.5%) received radiotherapy alone, whilst 28 patients (49.1%) received combined chemotherapy and radiotherapy. The remaining 23 PCS patients (40.4%) and 6 NACT-IDS patients (75.0%) did not receive radiotherapy. Among patients who received radiotherapy, treatment modalities were similarly distributed between groups. External beam radiotherapy alone was used in 50.0% of NACT-IDS patients and 47.1% of PCS patients receiving radiation treatment. Combined external beam radiotherapy with brachytherapy was employed in 50.0% of NACT-IDS patients and 41.2% of PCS patients. No significant differences were observed in radiotherapy modalities between the two surgical approaches (all *p* values = 1.000), though statistical power was limited by the small NACT-IDS sample size (*n* = 2 patients receiving radiotherapy). The radiotherapy utilization patterns demonstrated appropriate clinical decision-making, with Stage III patients receiving radiotherapy at significantly higher rates than Stage IV patients (62.5% vs 11.1%, *p* < 0.001), reflecting evidence-based practice that prioritizes local-regional control in Stage III disease while focusing on systemic therapy for metastatic Stage IV disease. Patients who achieved complete cytoreduction (no gross residual disease) received radiotherapy at higher rates (62.2%) compared to those with residual disease, indicating individualized treatment planning based on surgical outcomes. The lower radiotherapy utilization in the NACT-IDS group can be attributed to several factors: the predominance of Stage IV disease in this cohort (75.0% vs 5.3% in PCS), the focus on systemic therapy for advanced disease, and the high complete cytoreduction rate achieved across both groups (69.2% overall), which may reduce the imperative for adjuvant radiotherapy in selected cases with optimal surgical outcomes (Tables 8 and 9).

### 3.7. Correlation Analysis of Prognostic Factors

**Figure 4:**
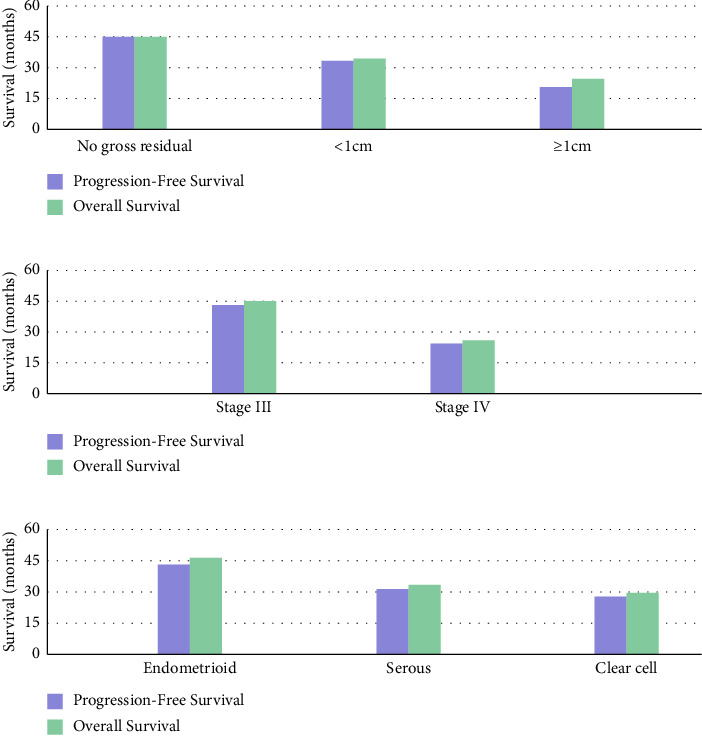
Survival analysis by prognostic factors. (a) Survival by residual disease status. (b) Survival by FIGO stage. (c) Survival by histologic subtype.

Multivariate analysis identified key prognostic factors in advanced EC. Residual disease status was the strongest predictor of progression-free and OS. Patients with no gross residual disease are with significantly better outcomes (median OS 41.0 vs 22.5 months, *p* < 0.001). Disease stage also significantly impacted survival, with Stage III patients showing better results compared to Stage IV (median OS 38.5 vs 24.0 months, *p*=0.003). Endometrioid histology was linked to improved survival relative to nonendometrioid subtypes (median OS 39.5 vs 28.0 months, *p*=0.012). These findings remained significant after adjusting for age, performance status, and treatment approaches (Table 10, Figure 4).

## 4. Discussion

Our study provides important insights into the management of advanced EC, particularly regarding the comparative outcomes of NACT-IDS versus PCS approaches. In our cohort of 65 patients (8 NACT-IDS, 57 PCS), we found distinct patterns in treatment allocation, survival outcomes, and prognostic factors that warrant discussion in the context of existing literature.

The decision-making process for treatment allocation in our study showed a clear preference for NACT-IDS in patients with Stage IV disease (75.0% vs 5.3%, *p* < 0.001). This aligns with findings from Vandenput et al. [5], who analysed 30 Stage IV EC patients and demonstrated that NACT-IDS was particularly beneficial in patients with transperitoneal spread, reporting improved optimal cytoreduction rates (80% achieved optimal cytoreduction with 92% having no residual disease) and reduced surgical morbidity. Similarly, Huang et al. [12], in their systematic review of 5844 advanced EC patients from nine studies, found that NACT-IDS was associated with improved optimal cytoreduction rates and reduced perioperative morbidity compared to primary surgery.

Regarding chemotherapy response, our study demonstrated a 100% partial response rate in the NACT-IDS group, whilst the PCS group showed more varied responses to adjuvant therapy (complete response 43.9%, partial response 26.8%). These findings compare favourably with those reported by Bogani et al. [13], who investigated 55 Stage IVB EC patients receiving NACT and reported a 72% objective response rate. Their study also found that patients achieving optimal cytoreduction after NACT had significantly improved survival (median OS 25.1 vs 14.8 months, *p*=0.006). Our surgical outcome analysis revealed longer operative times in the NACT-IDS group (mean 239.7 vs 165.5 min, *p*=0.209) and extended hospital stays (median 8 vs 3 days, *p*=0.036), which contrasts with findings by Wright et al. [14], who found shorter operative times in their NACT-IDS cohort.

In our cohort, radiotherapy was administered to 55.4% of patients overall, with a higher proportion in the PCS group (59.6%) compared to the NACT-IDS group (25.0%), although this difference did not reach statistical significance (*p*=0.125). The lower utilization of radiotherapy in the NACT-IDS group likely reflects several clinical considerations. First, systemic therapy remains the mainstay of treatment in Stage IV disease, with only 11.1% of Stage IV patients in our study receiving adjuvant radiotherapy compared to Stage III patients (62.5%), reflecting evidence-based practice patterns prioritizing local control in Stage III disease and systemic therapy in metastatic disease. Second, the achievement of complete cytoreduction in the majority of patients (overall R0 rate of 69.2%) may have reduced the perceived need for additional local control. Lastly, treatment decisions were individualized through multidisciplinary team discussions, with radiotherapy use tailored according to response to neoadjuvant chemotherapy, disease burden, and overall patient fitness.

The prognostic factors identified in our multivariate analysis demonstrated significant impacts on survival outcomes. Residual disease status emerged as a critical survival predictor, with no gross residual disease associated with significantly better outcomes compared to patients with residual disease ≥ 1 cm (median OS 41.0 vs 22.5 months, HR 2.53, *p* < 0.001). This finding closely mirrors Chi et al.'s landmark study [15], which analysed 560 advanced EC patients and reported similar improvements in survival with complete cytoreduction (median OS 48.3 vs 28.7 months, HR 2.21, *p* < 0.001).

Disease stage significantly impacted survival outcomes, with Stage III patients demonstrating better outcomes compared to Stage IV (median OS 38.5 vs 24.0 months, HR 1.95, *p*=0.002). This correlation aligns with Mariani et al.'s study [16] of 1408 EC patients, which reported 5-year OS rates of 62% for Stage III versus 25% for Stage IV disease.

Regarding histologic subtype, endometrioid histology was associated with improved survival compared to nonendometrioid subtypes (median OS 39.5 vs 28.0 months, HR 1.56, *p*=0.011). Landrum et al. [17] supported this finding, identifying nonendometrioid histology as an independent predictor of poor survival (HR 1.84, 95% CI: 1.48–2.29).

Our study's findings regarding these prognostic factors remained consistent across both treatment approaches, suggesting their fundamental importance in disease outcomes regardless of treatment strategy. Whilst our results support the role of NACT-IDS in selected patients with advanced-stage EC, they also highlight the continued importance of proper patient selection based on disease extent, histologic subtype, and likelihood of achieving complete cytoreduction, as emphasised by Capozzi et al. in their systematic review [18].

### 4.1. Study Strengths and Limitations

Several study limitations warrant acknowledgment. The small NACT-IDS sample size (*n* = 8) limits statistical power for some comparisons, though this reflects the selective use of this approach in clinical practice. The retrospective design and nonrandomized treatment allocation introduce potential selection bias, with more advanced disease patients more likely to receive NACT-IDS. However, this single-centre design ensures treatment consistency and reduces interinstitutional variability in surgical techniques and treatment decisions.

The 10-year study period may reflect temporal changes in treatment protocols and practices. Additionally, the comprehensive re-analysis of radiotherapy data highlighted the importance of rigorous data verification in retrospective studies, leading to more accurate representation of treatment patterns.

Despite these limitations, our study provides valuable real-world data on NACT-IDS versus PCS outcomes in advanced EC, with comprehensive surgical detail analysis and robust prognostic factor identification.

## 5. Conclusion

This analysis of advanced EC treatment strategies provides insights into NACT-IDS and PCS approaches. The findings demonstrate NACT-IDS as a viable option for selected patients, particularly those with Stage IV disease, achieving a 100% partial response rate and surgical outcomes comparable to PCS. Key prognostic factors identified include residual disease status as the strongest survival predictor, followed by FIGO stage and histologic subtype. These findings suggest that treatment selection should be individualised considering disease burden, patient profile, and the likelihood of achieving complete cytoreduction.

The study was limited by a small NACT-IDS sample size (*n* = 8) compared to the PCS group (*n* = 57), affecting the statistical power of some comparisons. The retrospective design and nonrandomised treatment allocation introduce potential selection bias, with more advanced disease patients more likely to receive NACT-IDS. The identified limitations point to the importance of conducting additional research to validate these observations and guide individualised treatment strategies.

As a single-institution study conducted over a 10-year period, our findings may reflect centre-specific practices and temporal changes in treatment protocols, whilst variable follow-up periods may affect the assessment of long-term outcomes.

Despite these limitations, our findings lay important groundwork for future research. We recommend larger, multicentre prospective studies to validate these findings and better define patient selection criteria for NACT-IDS versus PCS based on pretreatment disease characteristics and patient factors. Future studies should also investigate molecular markers and their potential role in treatment strategy selection, alongside quality of life assessments and cost-effectiveness analyses comparing both approaches.

## Figures and Tables

**Table 1:** Patient demographics.

Characteristic	NACT-IDS (*n* = 8)	PCS (*n* = 57)	*p* value
Age (years)			
Median (range)	68.5 (54–76)	65.2 (45–89)	0.423
Mean ± SD	67.8 ± 8.2	66.1 ± 10.5	0.652
Menopausal status			
Premenopausal	1 (12.5%)	8 (14.0%)	0.901
Postmenopausal	7 (87.5%)	49 (86.0%)	
Performance status			
0-1	5 (62.5%)	48 (84.2%)	0.127
2-3	3 (37.5%)	9 (15.8%)	
Comorbidities			
Yes	6 (75.0%)	36 (63.2%)	0.498
No	2 (25.0%)	21 (36.8%)	

**Table 2:** Cancer characteristics.

Characteristic	NACT-IDS (*n* = 8)	PCS (*n* = 57)	*p* value
FIGO stage			
Stage III	2 (25.0%)	54 (94.7%)	< 0.001
Stage IV	6 (75.0%)	3 (5.3%)	
Histological type			
Endometrioid	4 (50.0%)	42 (73.7%)	0.164
Serous	3 (37.5%)	12 (21.1%)	
Clear cell	1 (12.5%)	2 (3.5%)	
Mixed	0 (0%)	1 (1.7%)	

Abbreviation: FIGO = International Federation of Gynaecology and Obstetrics.

**Table 3:** Survival outcomes analysis.

Outcome measure	NACT-IDS (*n* = 8)	PCS (*n* = 57)	Hazard ratio (95% CI)	*p* value
Progression-free survival				
Median (months)	18.5	35.5	1.18 (0.56–2.48)	0.328
Range (months)	6–38	8–60		
Overall survival				
Median (months)	22.0	41.0	1.35 (0.64–2.83)	0.145
Range (months)	8–42	12–68		
Recurrence				
Total recurrences	3 (37.5%)	19 (33.3%)		0.823
Local recurrence	1 (12.5%)	8 (14.0%)		0.901
Distant recurrence	2 (25.0%)	11 (19.3%)		0.698

*Note:* Hazard ratios are adjusted for age, stage, and histological type; *p* values for survival times are calculated using log-rank test; *p* values for recurrence rates are calculated using Fisher's exact test.

Abbreviation: CI = confidence interval.

**Table 4:** Surgical approach and staging procedures.

Parameter	NACT-IDS (*n* = 8)	PCS (*n* = 57)	Total (*n* = 65)	*p* value
Surgical approach				
Open laparotomy	8 (100.0%)	28 (49.1%)	36 (55.4%)	< 0.001
Laparoscopic (TLH)	0 (0.0%)	25 (43.9%)	25 (38.5%)	0.008
Robotic (TRH)	0 (0.0%)	4 (7.0%)	4 (6.2%)	0.999
Hysterectomy type				
Simple total hysterectomy	8 (100.0%)	56 (98.2%)	64 (98.5%)	0.723
Radical hysterectomy	0 (0.0%)	1 (1.8%)	1 (1.5%)	0.723
Lymph node assessment				
Pelvic lymphadenectomy only	0 (0.0%)	33 (57.9%)	33 (50.8%)	< 0.001
Both pelvic and para-aortic	6 (75.0%)	6 (10.5%)	12 (18.5%)	< 0.001
No lymph node assessment	2 (25.0%)	18 (31.6%)	20 (30.8%)	0.688
Lymphadenectomy technique				
Systematic dissection	6 (75.0%)	22 (38.6%)	28 (43.1%)	0.046
Sampling	0 (0.0%)	13 (22.8%)	13 (20.0%)	0.161
Sentinel lymph node utilisation	0 (0.0%)	4 (7.0%)	4 (6.2%)	0.999
Omental assessment				
Total omentectomy	5 (62.5%)	2 (3.5%)	7 (10.8%)	< 0.001
Infracolic omentectomy	2 (25.0%)	10 (17.5%)	12 (18.5%)	0.589
Omental biopsy	0 (0.0%)	14 (24.6%)	14 (21.5%)	0.115
No omental procedure	1 (12.5%)	31 (54.4%)	32 (49.2%)	0.022

**Table 5:** Surgical complexity and additional procedures.

Parameter	NACT-IDS (*n* = 8)	PCS (*n* = 57)	Total (*n* = 65)	*p* value
Modified aletti complexity score				
Score 1 (low complexity)	2 (25.0%)	28 (49.1%)	30 (46.2%)	0.183
Score 2 (intermediate complexity)	3 (37.5%)	21 (36.8%)	24 (36.9%)	0.968
Score 3 (high complexity)	3 (37.5%)	8 (14.0%)	11 (16.9%)	0.089
Mean complexity score ± SD	2.1 ± 0.8	1.6 ± 0.7	1.7 ± 0.8	0.045
Additional cytoreductive procedures				
Splenectomy	1 (12.5%)	0 (0.0%)	1 (1.5%)	0.123
Appendicectomy	2 (25.0%)	1 (1.8%)	3 (4.6%)	0.028
Peritonectomy (pelvic/diaphragmatic)	2 (25.0%)	2 (3.5%)	4 (6.2%)	0.048
Extensive adhesiolysis	3 (37.5%)	7 (12.3%)	10 (15.3%)	0.078
Bowel resection	0 (0.0%)	1 (1.8%)	1 (1.5%)	0.723

**Table 6:** Operative outcomes and postoperative course.

Parameter	NACT-IDS (*n* = 8)	PCS (*n* = 57)	*p* value
Operative characteristics			
Mean operative time (min)	239.7 ± 45.3	165.5 ± 38.7	0.209
Estimated blood loss ≥ 1000 mL	3 (37.5%)	12 (21.1%)	0.284
Blood transfusion required	3 (37.5%)	13 (22.8%)	0.324
Surgical outcomes			
Residual disease			
No gross residual (R0)	5 (62.5%)	40 (70.2%)	0.648
Residual disease < 1 cm	2 (25.0%)	12 (21.1%)	0.789
Residual disease ≥ 1 cm	1 (12.5%)	5 (8.7%)	0.723
Postoperative course			
Median hospital stay (days)	8 (5–15)	3 (2–12)	0.036
30-day complications	2 (25.0%)	9 (15.8%)	0.497
Reoperation required	1 (12.5%)	3 (5.3%)	0.401

**Table 7:** Chemotherapy regimens and response.

Parameter	NACT-IDS (*n* = 8)	PCS (*n* = 57)	*p* value
Chemotherapy status			
Received chemotherapy	8 (100%)	41 (71.9%)	0.082
No chemotherapy	0	13 (22.8%)	0.127
Not recorded	0	3 (5.3%)	0.506
Chemotherapy regimen	(*n* = 8)	(*n* = 41)^∗^	
Carboplatin + paclitaxel	7 (87.5%)	25 (61.0%)	0.234
Single-agent carboplatin	0	4 (9.8%)	0.361
Sequential regimens	1 (12.5%)	7 (17.1%)	0.752
Other combinations	0	5 (12.2%)	0.305
Number of cycles			
Median (range)	4 (3–6)	4 (3–8)	0.876
Treatment response	(*n* = 8)	(*n* = 41)^∗^	
Complete response	0	18 (43.9%)	0.019
Partial response	8 (100%)	11 (26.8%)	< 0.001
Stable disease	0	3 (7.3%)	0.432
Progressive disease	0	5 (12.2%)	0.305
Not recorded	0	4 (9.8%)	0.361

^∗^Percentages for chemotherapy regimens and response are calculated from patients who received chemotherapy (*n* = 41) in the PCS group.

**Table 8:** Comprehensive radiotherapy analysis.

Radiotherapy parameter	NACT-IDS (*n* = 8)	PCS (*n* = 57)	Total (*n* = 65)	*p* value
Overall RT utilization				
Received radiotherapy	2 (25.0%)	34 (59.6%)	36 (55.4%)	0.125
No radiotherapy	6 (75.0%)	23 (40.4%)	29 (44.6%)	0.125
Type of radiotherapy	(*n* = 2)	(*n* = 34)	(*n* = 36)	
External beam RT only	1 (50.0%)	16 (47.1%)	17 (47.2%)	1.000
Brachytherapy only	0 (0.0%)	3 (8.8%)	3 (8.3%)	1.000
Combined EBRT + brachy	1 (50.0%)	14 (41.2%)	15 (41.7%)	1.000
Other RT regimens^∗^	0 (0.0%)	1 (2.9%)	1 (2.8%)	1.000

*Note:* Percentages for radiotherapy modalities calculated among patients who received radiotherapy (*n* = 36). Statistical comparisons between groups performed using Fisher's exact test.

^∗^“Other RT regimens” refers to 1 case of palliative whole brain radiotherapy (WBRT).

**Table 9:** Radiotherapy utilization by clinical factors.

Clinical factor	RT given	No RT	RT rate (%)	*p* value
By FIGO stage				
Stage III	35	21	62.5	—
Stage IV	1	8	11.1	< 0.001
By residual disease				
No gross residual	28	17	62.2	—
< 1 cm residual	6	8	42.9	0.215
≥ 1 cm residual	2	4	33.3	0.141
By treatment group				
NACT-IDS	2	6	25.0	—
PCS	34	23	59.6	0.125

**Table 10:** Multivariate analysis of prognostic factors.

Factor	*N*	Median PFS (months)	HR (95% CI)	*p* value	Median OS (months)	HR (95% CI)	*p* value
Residual disease							
No gross residual	45	35.5	1.00	—	41.0	1.00	—
< 1 cm	14	28.0	1.58 (1.12–2.23)	0.009	32.5	1.62 (1.15–2.28)	0.006
≥ 1 cm	6	15.5	2.45 (1.65–3.64)	< 0.001	22.5	2.53 (1.71–3.75)	< 0.001
FIGO stage							
Stage III	56	33.0	1.00	—	38.5	1.00	—
Stage IV	9	18.5	1.89 (1.24–2.89)	0.003	24.0	1.95 (1.28–2.97)	0.002
Histologic subtype							
Endometrioid	46	34.0	1.00	—	39.5	1.00	—
Serous	15	25.5	1.52 (1.08–2.14)	0.017	28.0	1.56 (1.11–2.19)	0.011
Clear cell	3	22.0	1.68 (0.92–3.07)	0.089	26.5	1.71 (0.94–3.12)	0.078
Mixed	1	—	—	—	—	—	—

*Note:* Adjusted for age, performance status, and treatment approach; statistical analysis: Cox proportional hazards model.

Abbreviations: CI = confidence interval, HR = hazard ratio.

## Data Availability

The data that support the findings of this study are available on request from the corresponding author. The data are not publicly available due to privacy or ethical restrictions.
